# Coordination of Oral Anticoagulant Care at Hospital Discharge (COACHeD): protocol for a pilot randomised controlled trial

**DOI:** 10.1186/s40814-022-01130-z

**Published:** 2022-08-02

**Authors:** Anne M. Holbrook, Kristina Vidug, Lindsay Yoo, Sue Troyan, Sam Schulman, James Douketis, Lehana Thabane, Stephen Giilck, Yousery Koubaesh, Sylvia Hyland, Karim Keshavjee, Joanne Ho, Jean-Eric Tarride, Amna Ahmed, Marianne Talman, Blair Leonard, Khursheed Ahmed, Mohammad Refaei, Deborah M. Siegal

**Affiliations:** 1 Division of Clinical Pharmacology and Toxicology, Department of Medicine, McMaster University, Hamilton, ON Canada; 2 Clinical Pharmacology Research, Research Institute of St Joes Hamilton, Hamilton, ON Canada; 3 Department of Health Research Methods, Evidence and Impact (HEI), McMaster University, Hamilton, ON Canada; 4 Division of General Internal Medicine, Department of Medicine, Faculty of Health Sciences, McMaster University, Hamilton, ON Canada; 5 Department of Medicine, Hamilton Health Sciences, Hamilton, ON Canada; 6 Divsion of Hematology and Thromboembolism, Department of Medicine, McMaster University, Hamilton, ON Canada; 7 Department of Medicine, Grand River Hospital, Kitchener, ON Canada; 8 Department of Medicine, Brantford General Hospital, Brantford, ON Canada; 9 Institute for Safe Medication Practices Canada, North York, ON Canada; 10 Institute of Health Policy, Management and Evaluation, University of Toronto, Toronto, ON Canada; 11 Research Institute for Aging, Schlegel-University of Waterloo, Waterloo, ON Canada; 12 Division of Geriatric Medicine, Department of Medicine, McMaster University, Hamilton, ON Canada; 13 Center for Health Economic and Policy Analysis (CHEPA), McMaster University, Hamilton, ON Canada; 14 Programs for Assessment of Technology in Health (PATH), Research Institute of St. Joe’s Hamilton, Hamilton, ON Canada; 15 Department of Medicine, Niagara Health System, Regional Municipality of Niagara, Canada; 16 Division of Hematology, Department of Medicine, University of Ottawa, Ottawa, ON Canada; 17 Ottawa Hospital Research Institute, Ottawa, ON Canada

**Keywords:** Anticoagulation, Medication management, Transitional care, Hospital discharge, Pilot trial

## Abstract

**Background:**

Oral anticoagulants (OACs) are commonly prescribed, have well-documented benefits for important clinical outcomes but have serious harms as well. Rates of OAC-related adverse events including thromboembolic and hemorrhagic events are especially high shortly after hospital discharge. Expert OAC management involving virtual care is a research priority given its potential to reach remote communities in a more feasible, timely, and less costly way than in-person care. Our objective is to test whether a focused, expert medication management intervention using a mix of in-person consultation and virtual care follow-up, is feasible and effective in preventing anticoagulation-related adverse events, for patients transitioning from hospital to home.

**Methods and analysis:**

A randomized, parallel, multicenter design enrolling consenting adult patients or the caregivers of cognitively impaired patients about to be discharged from medical wards with a discharge prescription for an OAC. The interdisciplinary multimodal intervention is led by a clinical pharmacologist and includes a detailed discharge medication reconciliation and management plan focused on oral anticoagulants at hospital discharge; a circle of care handover and coordination with patient, hospital team and community providers; and early post-discharge follow-up virtual medication check-up visits at 24 h, 1 week, and 1 month. The control group will receive usual care plus encouragement to use the Thrombosis Canada website.

The primary feasibility outcomes include recruitment rate, participant retention rates, trial resources management, and the secondary clinical outcomes include adverse anticoagulant safety events composite (AASE), coordination and continuity of care, medication-related problems, quality of life, and healthcare resource utilization. Follow-up is 3 months.

**Discussion:**

This pilot RCT tests whether there is sufficient feasibility and merit in coordinating oral anticoagulant care early post-hospital discharge to warrant a full sized RCT.

**Trial registration:**

NCT02777047.

**Supplementary Information:**

The online version contains supplementary material available at 10.1186/s40814-022-01130-z.

## Strengths and limitations of this study

- There is a lack of high-quality evidence on hospital discharge strategies which reduce oral anticoagulant (OAC)-related adverse events.
- Our methods build on our prior research showing that early post-hospital discharge is a high-risk period for adverse events for patients, that coordination of OAC management peri-hospital discharge is hypothesized to reduce events, and that virtual care may be more cost-effective than in person care.
- This pragmatic pilot randomized trial combines expert multidisciplinary medication management led by Clinical Pharmacology at hospital discharge with virtual care follow-up.
- Our feasibility outcomes introduce the concept of research resource utilization and management, expressed as cost per patient completing the trial, as a key outcome.

## Introduction

### Background and rationale

Institute for Safe Medication Practices (ISMP) data suggest that approximately 300,000 Canadians suffer serious, disabling, or fatal medication-related harm annually [1, 2]. Anticoagulants are the most common cause of medication-related serious harm, in terms of emergency department visits, hospitalizations, and fatalities [3, 4]. Transitions in care have been identified as a particularly high-risk period for adverse events [5]. Each adverse drug event requiring a hospital visit approximately doubles the cost of care in the subsequent 6 months [1]. Indeed, our previous study of thromboembolic and hemorrhagic events after hospital discharge for patients taking oral anticoagulants (OACs) found rates to be approximately 2 to 3 times higher in the first month compared to later [6]. Root cause analyses and patient safety inquiries cite problems with recognition of individual risk factors for benefit versus harm, drug interactions and contraindications, dosing adjustments over time and around procedures, drug monitoring and reversal strategies, and with communications and poor adherence by patients [7–9]. Furthermore, the direct oral anticoagulants, each with different dosage regimens based on indication and no widely available test to measure the anticoagulant effect, have increased opportunities for medication errors, while greatly increasing drug costs [2, 10, 11].

Anticoagulants are a high priority drug family for improved medication management because of (a) established benefits to reduce the risk for stroke due to atrial fibrillation by approximately 70%, the risk for recurrent venous thromboembolism by more than 90%, and the risk of death by approximately 25%; (b) widespread utilization with more than 12% of people >85 years of age taking OACs and more than 7 million prescriptions dispensed annually in Canada; and (c) ongoing under-prescribing of OACs for eligible patients, over-prescribing of reduced doses off-label, and use of inferior treatments such as aspirin [12–15].

Although there are several well-developed anticoagulation management guidelines with detailed, evidence-informed recommendations, it is difficult for physicians and other health care providers to keep up with evolving evidence, and many patient situations lack high-quality evidence to support a specific approach [16–18]. Thrombosis Canada provides updated, user-friendly succinct clinical guidance for physicians and patients with point-of-care decision support tools; however, the value of supplemental patient education and patient decision aids to improve outcomes remains unproven [19].

Optimal medication safety requires not only best practices based on clinical evidence but also impeccable application and uptake. The latter requires coordination, communication with and education of all key providers, caregivers, and the patient, as well as frequent monitoring, handovers, and constant quality improvement [20–22]. All of these may be most efficiently provided on a large scale across large regions by telehealth or virtual visits [23–25]. However, the effectiveness and cost-effectiveness of telehealth or online interventions for medication management remain uncertain [26, 27]. In addition, methods of coordination and communication of care, including in anticoagulant management programs or clinics vary, although toolkits exist for content guidance [28–30]. Significantly, coordination interventions have rarely involved clinical pharmacologists—medical specialists with expertise in medication management and the ability to diagnose, prescribe, and change prescriptions.

We hypothesize that expert coordination and management of OAC therapy combined with frequent virtual visits by a pharmacist and clinical pharmacologist in the early post-hospital discharge period, plus regular communication with the patient’s circle of care, could decrease adverse anticoagulant-related events and associated healthcare resource utilization while improving patient’s health-related quality of life compared to usual care.

### Objectives

The aim of this pilot randomized controlled trial (RCT) is to test whether high-quality, easily scalable, expert multidisciplinary medication management, and care coordination at hospital discharge and during short-term post-discharge virtual visits are feasible (primary) and can improve oral anticoagulant-related adverse event (thrombotic events, bleeds, and deaths) rates, medication problems, quality-of-life, cost-effectiveness, and satisfaction with care (secondary) during a high-risk transition in care period.

## Methods and analysis

### Trial design and setting

The protocol was developed according to the SPIRIT and TIDieR guidelines [31, 32]. This pragmatic pilot RCT is designed as a 2-arm, parallel, blinded assessment, variable block randomized trial with individual level randomization and outcomes of feasibility and clinical outcomes, during 3 months of follow-up.

Participating sites include 6 hospitals in Southwest Ontario—3 academic teaching hospitals and 3 community hospitals. The trial was originally scheduled to begin in late 2019 but was delayed initially by staff shortages, then by COVID-19 restrictions on hospital-based research [33].

### Eligibility criteria

Inclusion criteria include (a) adult patients within a day of their hospital discharge from internal medicine services with a discharge prescription for an OAC intended to be taken for at least 4 weeks (including both incident and prevalent users), (b) discharge is to home or to a congregant setting such as retirement home where the patient manages their own medications, (c) English-speaking, and (d) capable of providing informed consent. To ensure that we can ethically recruit vulnerable participants including those cognitively impaired, the ability to consent will be measured by the COACHeD Capacity to Consent test, requiring a score of 14 or more (Additional file 3) [34]. If the patient does not pass, a close caregiver (defined as a family member in daily contact with the patient and involved in their medication supervision) will be invited to provide consent on the patient’s behalf by signing a caregiver consent form.

Patients will be excluded if they are less than 18 years of age and have an expected lifespan of less than 3 months and will be discharged to long-term care or other institution where medications are controlled by staff or decline informed consent.

### Intervention

Figure 1 shows the study flow diagram including time and activities for both groups (see Additional file 1).

The Intervention Arm includes the following:

- Interdisciplinary intervention led by a clinical pharmacologist who is a leader in evidence-based prescribing—includes a detailed discharge medication reconciliation and management plan focussed on oral anticoagulants at hospital discharge; a circle of care handover and coordination with patient, hospital team, and community providers; three scheduled early post-discharge virtual medication check-up visits at 24 h, 1 week, and 1 month with triage of any problems. Medication reconciliation reviews hospital-administered medications compared to pre-admission medications [35]. Medication management is the more complex task of assessing and revising medications in light of the individual patient’s diagnoses, current symptoms and signs, risk factors, allergies and intolerances, other medications, and goals. In this study, all medications will be reviewed with a focus on OAC choice, dosage, indication, duration, potential drug interactions, patient risk factors for thromboembolism versus bleeding, drug insurance, adherence challenges, and health literacy. A study pharmacist with additional training will complete the detailed medication reconciliation then confer with the clinical pharmacologist who will lead the medication management including meeting with the patient and making prescription changes.
- Hand-overs to the community care team including the main patient caregiver (if applicable), family physician, medical specialist(s), and community pharmacist, using a templated consult summary note which includes a patient profile, details of recent hospitalization, discharge medications, an OAC monitoring checklist, circle of care and upcoming appointments, and recommendations. Figure 2 shows an example consult note (see Additional file 2)*.* The monitoring is based on (a) best evidence (updated guidelines and dedicated evidence review using the CLOT repository of CanVECTOR and McMaster’s Health Information Research Unit), and decision aid content for patients and their families to assist in anticoagulant knowledge and adherence; (b) best practices regarding discharge medication management, virtual care, scalable coordination of care with clear accountability, communication, and teletriage where situations require medical intervention [5, 16, 19, 36–40]. All consult notes are reviewed in detail with the Clinical Pharmacologist.
- “Virtual visits” (secure video calls from within our electronic medical record (EMR) or phone visits where video is not possible) by the study pharmacist at three follow-up time points—24 h post-discharge to ensure the discharge prescription medications were obtained and understood, review the OAC monitoring checklist, review other medications, and solicit concerns; and at 1 week and 1 month to ensure medication adherence, review the OAC monitoring checklist and other medications, and solicit concerns. After each follow-up visit, a summary consult note will be sent to all circle of care providers, and any clinical events or serious concerns will be addressed by the Clinical Pharmacologist or directed to patient’s family physician via phone call or direct email. Each follow-up visit with intervention patients will be recorded and tracked to ensure adherence to protocols.
- Teletriage—The patients have the study pharmacist’s contact information and can phone for assistance at any time. The study pharmacist is in constant communication with a Clinical Pharmacologist investigator for guidance. An expert thrombosis specialist will be available on call as needed.

Control Arm: Patients allocated to the control group will receive usual care, plus the URL to Thrombosis Canada website. Usual care in the participating sites includes OAC management by family doctors except for new thromboembolic events which will be followed short term by thromboembolism or hematology specialists, complicated atrial fibrillation which will have cardiology involved temporarily, and a small proportion of warfarin management which is provided in an anticoagulation clinic. This choice of the control group is the most relevant for generalizability to both academic and community practices.

For both arms, there are no restrictions placed on concomitant medical intervention or treatments, as this is a pragmatic randomized trial.

### Outcomes

Study outcomes, their measurement methods, timing, and analysis are presented in detail in Table 1 and Table 2 and include the following:

1. Primary outcomes, which are study feasibility outcomes, include recruitment and retention rates, and estimated resources required per patient to complete the main trial. Feasibility will be achieved if we can recruit at least 30% of those eligible, retain 90% of those recruited, and spend no more than $1500 per patient running the trial. Secondary feasibility outcomes include barriers and facilitators to success of the primary outcomes, in terms of process and management issues. These will be used to determine whether a large definitive research study is likely to be feasible, taking into account the practical aspects of managing and funding the project [41, 42].
2. Secondary clinical outcomes include the following:
  - The Adverse Anticoagulant Safety Events composite (AASE) which is any of thromboembolic events or clinically relevant bleeding or death. Thromboembolic events include objectively verified ischemic stroke, systemic embolism, pulmonary embolism, or DVT. Clinically relevant bleeds in this study is defined as bleeding that causes death, hospitalization, or emergency department visits.
  - Coordination and Continuity of Care: Adapted from Health Quality Ontario’s draft guidance and a Rand instrument, the Coordination and Continuity of Care Questionnaire is designed to measure the quality of the transitional and follow-up care [5, 43, 44].
  - Patient Quality of Life: The EQ-5D-5L is the 5-level classification system of the EQ-5D, a measure of health status from the EuroQol group [45]. Using EQ-5D-5L, respondents are asked a short series of questions about mobility, self-care, usual activities, pain discomfort, and anxiety/depression, as well as a summary visual analogue scale. This scale, which provides utility measurements, has been well validated for the Canadian population [45–47].
  - Patient Knowledge of OAC Management: Insufficient patient knowledge about OAC may predict poor medication adherence and inadequate anticoagulation control [48]. The COACHeD OAC Knowledge Questionnaire tests knowledge of the therapeutic objective, process of use, safety, and maintenance of the medications [44].
  - Satisfaction with Care: Satisfaction reported by patients and by key health professionals is one of the recommended outcomes to report in medical research, as it may influence adherence [49, 50]. This outcome will be assessed by the Patient/Caregiver Study Satisfaction Survey and by the Provider Study Satisfaction Survey [44].
  - Medication Problems: We will be assessing problems with appropriateness, with medication errors characterized using the National Coordinating Council for Medication Error Reporting and Prevention (NCC-MERP) scale and with medication adherence and attitudes as measured by COMPETE Medication Problems Questionnaire [51, 52].
  - Resource utilization: This is a key outcome to determine cost-effectiveness and cost-utility which then determines whether health care systems might pay for this type of care [53, 54]. We will be measuring all types of health care utilization by patients [43, 46, 54].

**Table 1:** Study outcome assessment

Outcomes^**a**^		Variable type	Criteria for success	Outcome measure	Method of analysis
**A. Feasibility outcomes**					
Primary feasibility outcomes	Participant recruitment rates	Continuous (%)	≥ 30% of those eligible will be considered success	Percentage recruited and rate of recruitment (of those screened, of those eligible, and of those approached). Percentage of those recruited (signed informed consent) who complete the end of study assessment at 3 months.	Count of percentages and rates over time
	Participant retention rate	Continuous (%)	≥ 90% is considered success		
	Study resource utilization	Continuous ($CND)	< $1500 per patient recruited and completing the study	Study costs per patient recruited	Cost of personnel, supplies, travel, etc., to run the study/# patients recruited
Secondary feasibility outcomes	Process assessments Management assessments Scientific assessments	Mix of continuous, categorical, and qualitative data	Implementation is successful and feasible, questionnaires are acceptable, and data gathered are reliable and valid	Detailed questions in feasibility assessment	Descriptive
**B. Clinical outcomes**	**Hypothesis**				
Primary clinical outcome	Adverse anticoagulant safety event composite (AASE)	Continuous (%)	Intervention group will have fewer AASE compared to the control group	Count of AASE at the end of the study (3 months). Rates will be expressed as mean rate per patient-year by research assistant.	Chi-square test
Secondary clinical outcomes	Coordination and continuity of care	Continuous	Intervention will have higher ratings than control	Coordination and continuity of care questionnaire partially administered post-discharge and finalized at study end (3 months) by telephone.	*T* tests
	Patient quality of life	Continuous	Intervention will improve more than control	EQ5D-5L—difference in mean change scores at the end of the study.	*T* tests
	OAC management knowledge - patient	Continuous	Intervention will have higher scores than control	OAC knowledge questionnaire	*T* tests
	Satisfaction with care (providers & patients)	Continuous	Intervention will have higher scores than control	Satisfaction questionnaires	Descriptive analysis
	Medication Problems	Continuous	Intervention will have fewer medication problems than control	Composite of OAC-APEQ, COMPETE adherence, and medication error scores	*T* tests
	Cost-effectiveness	Continuous	Intervention will be cost-effective compared to control using a threshold of $50,000 per QALY.	Cost per AASE avoided and cost per quality-adjusted life-years (QALYs)	Economic analysis
	Individual Clinical Events: a) Bleeding events (clinically relevant), b) Thromboembolic events (all); c) Deaths; d) Hospitalizations or ED visits	Continuous	Intervention will have lower event rates than control	Event rate counts from end-study interviews	*T* tests

**Table 2:** COACHeD schedule of events

Assessment	Enrolment		Study in-progress			Study end
	Baseline	Hospital discharge	24h	1Week	1 month	3 months
Eligibility screen	A					
COACHeD Brief Assessment of Capacity to Consent	B					
Informed consent	1/C					
Coordination Continuity of Care Questionnaire (CCCQ)	I/C					I/C
EQ-5D-5L v10-Canada	1/C					I/C
OAC knowledge test	I/C					I/C
Randomization and allocation	I/C					
Discharge medication reconciliation and management, circle of care communication and coordination		I				
Virtual Visit Call #1			I			
Virtual Visit Call #2				I		
Virtual Visit Call #3					I	
Adverse anticoagulant safety events (AASE)						I/C
AASE individual components						I/C
Patient/Caregiver Satisfaction Questionnaire						I/C
Provider Satisfaction Questionnaire						I/C
Health Resource Utilization Questionnaire						I/C
Medication-related problems						I/C

*A*, all potentially eligible patients; *B*, all eligible and interested patients; *I*, intervention patient; *C*, control patient; *I/C*, both groups

### Sample size estimation

Pilot RCT guidance suggests that where sample size is calculated, it be based on the ability to detect a significant feasibility problem that might interfere with a subsequent full-size RCT—for example, difficulty with recruitment and retention, accuracy of outcome event capture and adjudication, frequency of events, and costing suggesting the intervention is not remotely cost-effective [55]. If a feasibility problem exists at a rate of 5% probability, it can be identified with 95% confidence with a sample of 59 participants [55].

### Recruitment methods

Background work flow studies at several of the participating hospitals have confirmed that patient discharge is a complex, hurried process that is often rescheduled multiple times because of fluctuations in patient health or delays in required tests, procedures, or milestones. For feasibility reasons, we plan to use a rolling recruitment method, spending 2 weeks at each hospital on a cyclical basis until a quota is recruited (10–12 patients per site). Rounds and recruitment posters will advertise the study; in some locations, the EMR can assist with screening criteria, and in all locations, research staff are seeking eligible patients in consultation with the Most Responsible Physician team personnel.

### Allocation

Participants who meet all the inclusion/exclusion criteria at screening and have completed informed consent will be enrolled in the study, complete baseline assessments, then will be randomized via a computer-generated randomization sequence stratified by site to one of the two study arms, intervention, or control.

A statistician will prepare the randomization schedule using an adaptive biased-coin strategy [56]. Randomization will be stratified by site to maintain balance and minimize the predictability of treatment assignments [57]. To restrict the treatment group imbalance, a maximal tolerable imbalance between treatment groups will be incorporated into the schedule [58]. The randomization schedule will be produced by a program written in SAS V9.4 software (SAS Institute Inc., Cary, NC, USA) and implemented in REDCap on a centralized computer where each patient’s treatment assignment will be available on-line to the research pharmacist at the time of randomization. This process ensures allocation concealment, and randomization awareness where necessary, for example, for the intervention staff.

### Blinding

Since this is a pragmatic RCT of care coordination, it will not be possible to completely blind patients or their providers; however, outcome data collectors, adjudicators, and statisticians will be blinded to group allocation until analysis is completed at the end of the study.

### Data collection methods

Trained research staff will conduct the interviews with the patients or caregivers, entering data electronically on study laptops directly into REDCap case report forms. The participants’ medical records will be reviewed to abstract data on baseline characteristics, medical history, and medication information. Strategies to promote participant retention and complete follow-up include reminding participants in advance of their end-of-study visit and communicating by email if email address is provided at baseline. Participants who drop out of the study will have their data to that point retained in the study, as approved by REB, to avoid bias. The reasons for study non-completion will be recorded.

### Data management

REDCap (Research Electronic Data Capture) is our study software platform—secure, web-based, providing interfaces for validated data capture, role-specific access, audit trails for tracking data manipulation and exports, automated export procedures to SAS and encrypted transmissions [59, 60]. Paper study documents including signed informed consent forms will be stored in our secure research office once they are scanned into REDCap study files. Regular data quality checks, such as automatic range checks, will be performed by the study team to identify data that appear inconsistent, incomplete, or inaccurate.

Patients are not identifiable in the project results database. The identifying information required for the clinical team to deliver the intervention is kept in a separate database. Access to the final dataset will be restricted to the core research team.

### Statistical analyses

The reporting of the results of this trial will follow the CONSORT extension to pilot trials [41]. We will use descriptive statistics for presentation of baseline variables and adequacy of follow-up. Feasibility analysis including recruitment rate (≥30% is considered success), participant retention rate (≥90% to end of study is considered success), study resource utilization required (CAD $1500 per patient recruited and completing the study is considered a threshold), management assessment, and scientific assessment will be descriptive.

Analysis will use intention-to-treat methods with censoring only if the patient dies or drops out of the study with refusal of negotiated further assessments. A sensitivity analysis of the subgroup of patients who received all 3 planned follow-up intervention calls and completed the end-study data collection (per protocol analysis) will be carried out. Research staff and statisticians will review outcome data and analysis blinded to group identification.

The primary clinical endpoint will be the incidence of adjudicated AASE during the follow-up period, using proportions, and chi-square testing. Secondary endpoint analyses of the coordination and continuity of care, patient quality of life, OAC management knowledge satisfaction with care (providers & patients), and resource utilization will be analyzed using *t* tests. The incidence of the adjudicated individual clinical events (clinically relevant bleeding events, thromboembolic events, all-cause hospitalizations, and emergency department visits) will be analyzed using the methods described above for AASE. Public unit costs from Ontario will be used to cost healthcare resource utilization collected as part of the trial. Using an area under the curve approach, quality-adjusted life years (QALYs) will be determined by weighting the EQ-5D-5L health utility scores by time spent in health state. Costs and outcomes (i.e., QALYs, AASE) between the interventions will be compared from a public payer perspective. Given the short follow-up, a low risk of the trial and pilot design, no interim analysis or imputation for missing data is planned. All analyses will be performed using SAS V9.4 software (SAS Institute Inc., Cary, NC, USA).

### Data monitoring

Since this trial does not involve any investigational product or procedure, and uses the main anticoagulant safety events and medication safety as outcome events, a formal Data Safety Monitoring Board is not required. Any serious adverse event will be reviewed by our Trial Steering Committee (TSC) within a week of detection, to discern any attribution to our procedures. If found to be due to our coordination procedures, the trial steering committee will recommend whether modifications are indicated. The TSC will be composed of individuals with expertise in clinical trials, chaired by the lead statistician, include the PI, the operational statistician plus a methodologist independent of the study team. Similarly, since this is a short pilot pragmatic RCT where no harm is expected and adjustment of trial procedures may be necessary for feasibility, no formal external auditing of trial conduct is planned. There is no requirement for additional ancillary and post-trial care for those who might come to harm while in the trial, as usual medical care which covers this eventuality is already in place.

### Trial management

The trial Principal Investigator will be responsible for communicating any changes to the study, new information, or unanticipated events to the REB, to the sponsor, and to Local Principal Investigators (LPI). The LPI (also called Site Investigator) is responsible for supervising any individual or party to whom they have delegated tasks at the trial site. A Trial Management Group (TMG) will be responsible for the day-to-day management of the trial and will include at a minimum the trial PI, the local PIs, and the trial Research Coordinator. The group will closely review all aspects of the conduct and progress of the trial, ensuring that there is a forum for identifying and addressing issues. Particular attention will be paid to progress towards trial milestones, adherence to the protocol and good research practices. Meetings will be minuted and retained in the study REDCap database.

### Patient contributions

Patients were specifically involved in the planning of this trial in several ways. A patient representative is a co-investigator (K.A.). In preparation for this trial, we investigated barriers and facilitators to optimal OAC management, using a systematic review of the literature and then a qualitative focus group study of the opinions of 26 patients/caregivers and 16 providers [61, 62]. These influenced the intervention content and timing. In addition, our choice of outcomes was guided by our recently published systematic survey of the literature regarding patient-important outcomes in OAC trials, as advised by patient groups [63]. One of our Knowledge User—dissemination lead groups is ISMP Canada who are the national leads on medication safety with well developed 2-way communications with patients.

### Ethics and dissemination

The study has been approved (study #1639) by the Hamilton Integrated Research Ethics Board, Brant Community Healthcare System Research Ethics Committee, and by the Tri-Hospital Research Ethics Board of Waterloo. Significant protocol modifications will be proactively communicated to the research ethics boards through study amendments to obtain approval prior to the changes being implemented. Each modification will be assessed to determine whether it warrants communication with trial participants.

Dissemination will be through presentations, publications, our research social media, incorporation into our knowledge partner communications to stakeholders, and possible future grant application. The results of the trial will be reported first to trial collaborators. The main report will be drafted by the trial coordinating team, and the final version will be agreed by the Trial Steering Committee before submission for publication, on behalf of the collaboration. The trial will be reported in accordance with the Consolidated Standards of Reporting Trials (CONSORT) guidelines (www.consort-statement.org). The results of the trial will be shared widely, and participants are able to request a copy of the results through contacting the local study team.

## Supplementary Information

**Additional file 1: Figure 1**. COACHeD Flow Diagram.

**Additional file 2: Figure 2**. Sample COACHeD Consult at Discharge.

**Additional file 3.** COACHeD Capacity to Consent Questionnaire.

## Data Availability

An anonymized dataset will be shared in accordance with future requirements of our funders, the Canadian Institutes of Health Research.

## Abbreviations

AASE: Adverse anticoagulant safety events
COACHeD: Coordination of oral anticoagulant care at hospital discharge
CONSORT: Consolidated standard of reporting trials
DVT: Deep vein thrombosis
EMR: Electronic medical records
EQ-5D-5L: European Quality of Life Five Dimension
ISMP: Institute for Safe Medication Practices
LPI: Local principal investigator
NCC-MERP: National Coordinating Council for Medication Error Reporting and Prevention
OAC: Oral anticoagulant
PI: Principal investigator
QALY: Quality-adjusted life years
RCT: Randomized control trial
REB: Research ethics board
REDCap: Research electronic data capture
TMG: Trial management group
TSC: Trial steering committee
