# Supplementary figures and images for: Coordination of Oral Anticoagulant Care at Hospital Discharge (COACHeD): protocol for a pilot randomised controlled trial
Source: Pilot Feasibility Stud. 2022 Aug 2;8:166. doi: 10.1186/s40814-022-01130-z (PMC9344454; doi:10.1186/s40814-022-01130-z)

**Figure 1. COACHEd Flow Diagram**

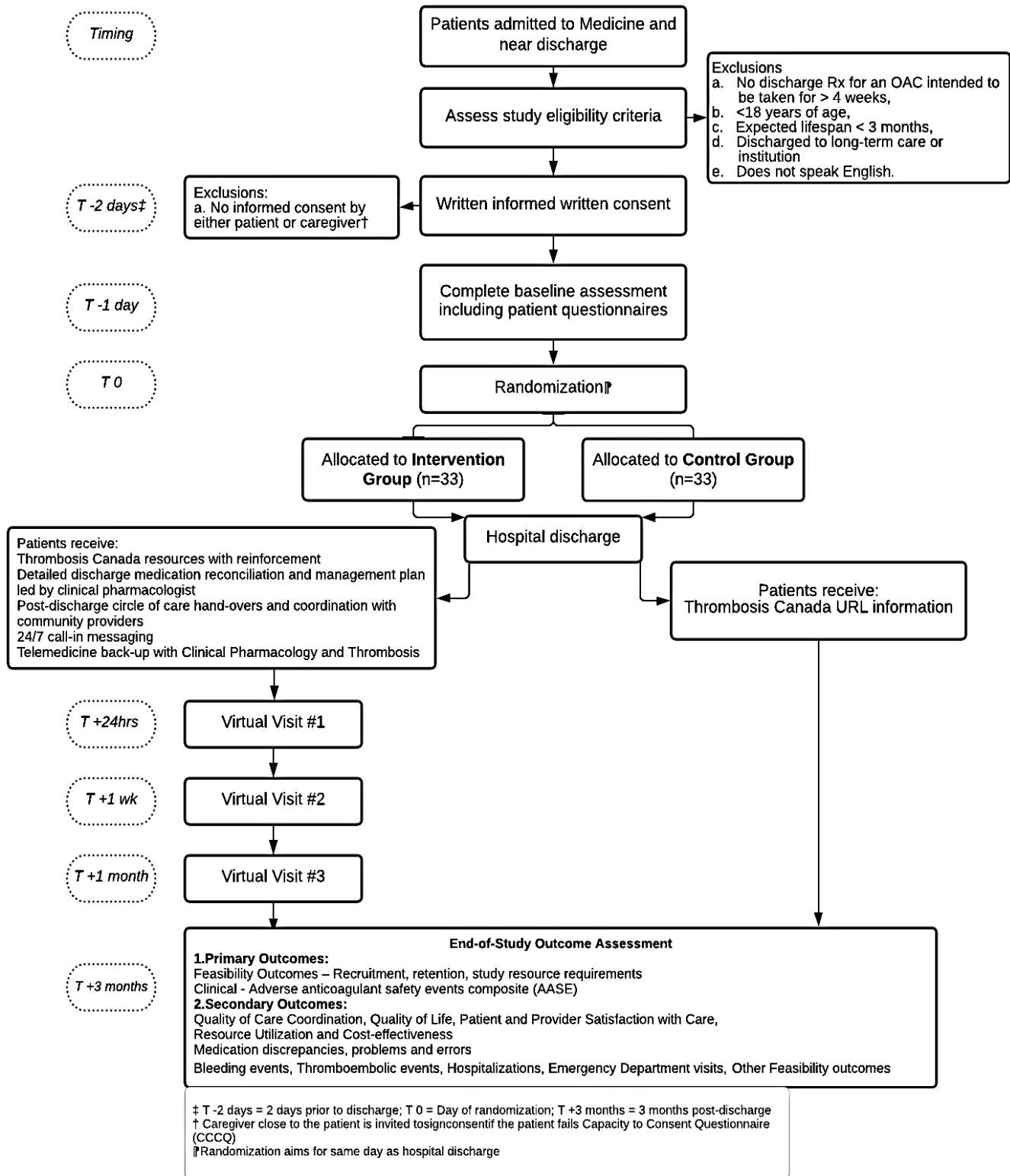

Supplement: Supplementary file 1 — Additional file 1: Figure 1. COACHeD Flow Diagram. [file 40814_2022_1130_MOESM1_ESM.pdf]
