# Supplementary material for: Coordination of Oral Anticoagulant Care at Hospital Discharge (COACHeD): protocol for a pilot randomised controlled trial
Source: Pilot Feasibility Stud. 2022 Aug 2;8:166. doi: 10.1186/s40814-022-01130-z (PMC9344454; doi:10.1186/s40814-022-01130-z)
Supplement: Supplementary file 3 — Additional file 3. COACHeD Capacity to Consent Questionnaire. [file 40814_2022_1130_MOESM3_ESM.docx]

**Additional file 3. COACHeD Capacity to Consent Questionnaire**

| **Question to Participant** | **Score** | **Guide to Scoring ⁋** | |
| --- | --- | --- | --- |
| 1. What is the purpose of the study that was just described to you? | 0  1  2 | 0 = I don’t know/ No clue  1 = Partial response regarding medication/treatment (without mention of medication management/coordinated care)  2 = (*One of the below or similar*):   - Coordinate care or communication from hospital to home - Improve medication safety or medication management - Better communication with my providers about my medications - Help prevent complications related to anticoagulants (blood thinners) |  |
| 1. What aspects of the study described might encourage you to participate? | 0  1  2 | 0 = I don’t know  1 = Partial response regarding treatment, follow-up  2 = (*At least one of the specific themes below, or similar*):   - Medication review with the pharmacist - Help me understand what is going on with my medications - Avoid confusion about my medications when I get discharged back home - Get advice about my medications and have my questions answered - Better communication with my doctors and specialists, so that they understand what happened and what is going on with my medications |  |
| 1. Do you believe this study is primarily for research or primarily for treatment? | 0  2 | 0 = Treatment, or don’t know  2 = RESEARCH |  |
| 1. Do you have to be in this study if you do not want to participate? | 0  2 | 0 = Yes or don’t know  2 = NO |  |
| 1. If you withdraw from this study, will you still be able to receive your regular treatment? | 0  2 | 0 = No  2 = Yes |  |
| 1. Can you tell us a couple of things that you will be asked to do If you participate in the study? | 0  1  2 | 0 = I don’t know/no correct response  1 = Only one of the below  2 = (*At least **two** of the following or similar*):   - Go through my medications with the pharmacist, - Answer some questionnaires at the beginning of the study, - Participate in phone visits with pharmacist about my medications at different time points; - Answer questions at the end of study |  |
| 1. What might be some risks or downsides involved with participating in this study? | 0  1  2 | 0 = I don’t know  1 = Partial response with generalities (eg, ‘waste of time’, ‘too many phone calls’, etc  2 = (*One of the themes below or similar*):   - Inconvenience or hassle of research coordinators phoning me at three different time points - Time commitment involved at the beginning or with the phone calls - Potential for privacy breach of my personal health information - Too many people involved in my care |  |
| 1. Can you describe some of the benefits that you may gain by participating in this study? | 0  1  2 | 0 = I don’t know  1 = Partial response on only one of the themes below  2 = (*At least one of the below or similar*):   - Avoiding complications from my anticoagulant - Help from a pharmacist with managing my medications, - Understanding my medications better, - Getting advice on what to do after I go home from the hospital, - Better communication with my doctors about what is going on with my medications |  |
| 1. Is it possible that being in this study will not have any benefit to you personally? | 0  2 | 0 = No, or don’t know  2 = YES |  |
| 1. Who would pay for your medical care if you came to any harm as a direct result of participating in this study? | 0  1  2 | 0 = I have no idea; I will have to pay;  1 = don’t know or not sure; someone else will pay (possibly the hospital or research program)  2 = No change; OHIP, government would pay for any medical care. There would be no cost to me |  |
