# Supplementary material for: Coordination of Oral Anticoagulant Care at Hospital Discharge (COACHeD): protocol for a pilot randomised controlled trial
Source: Pilot Feasibility Stud. 2022 Aug 2;8:166. doi: 10.1186/s40814-022-01130-z (PMC9344454; doi:10.1186/s40814-022-01130-z)
Supplement: Supplementary file 2 — Additional file 2: Figure 2. Sample COACHeD Consult at Discharge. [file 40814_2022_1130_MOESM2_ESM.pdf]

St. Joseph's Healthcare Hamilton

Division of Clinical Pharmacology & Toxicology

Clinical Pharmacology & Toxicology — Department of Medicine

<u>To</u>: Dr. Family Doctor | <u>Date</u>: xx/xx/2022

<u>Address</u>: 123 ABCD Avenue <u>Tel</u>: 905-222-1111 | <u>Fax</u>: 905-333-4444

<u>Patient</u>: Patient Patient

<u>Address</u>: 111 XYZ Street | <u>DOB</u>: 01/01/1952 | <u>MRN:</u> H0000111 | <u>HCN</u>: 1111222333

<u>Community Pharmacy</u>: Pharmacy Drug Mart, Road Rd, Hamilton ON. | <u>Phone</u>: 905-111-2222 | <u>Fax</u>: 905-111-2222

Dear Dr. Family Doctor,

Mrs. Patient is a 69 year-old female who has consented to enrol in a randomized trial called "*Coordination of Oral Anticoagulant Care at Hospital Discharge (COACHeD)*". This study is led by Dr. Anne Holbrook, a Clinical Pharmacology and Internal Medicine specialist, along with Thrombosis and other specialists. This visit was reviewed and the note was revised for important content by Dr. Holbrook.

Mrs. Patient was admitted to St. Joeseph's Hospital Hamilton on Day month, 2021 for pulmonary embolism. This was an unprovoked episode of PE, her first thromboembolic event and was marked by pleuritic pain only without hemodynamic compromise. She has no diagnosis of cancer.

She has a history of hypertension, type 2 diabetes, and insomnia. She was discharged on Day month, 2021. She has been newly started on Rivaroxaban 15mg po bid for 21 days followed by 20mg po daily thereafter. I spoke with Mrs. Patient pre-discharge and also 1-day post-discharge, with a focus on the oral anticoagulant (OAC) management issues. The following is a current medication list, and summary of our discussion:

1. Current Medication List

   <u>Prescription</u>

   i. Rivaroxaban 15mg po bid (**New**)
   ii. Janumet 500/50mg po BID (*dose decreased from pre-admission*)
   iii. Lorazepam 1 mg po hs
   iv. Perindopril 8 mg po daily

   <u>Over- the-Counter</u>: None

   <u>Recent Discontinuations</u>

   i. Janumet 1000/50mg po BID
   ii. Amlodipine 5mg po daily

2. Anticoagulant-Related Items

   a. Dosing of Rivaroxaban: The dosing of Rivaroxaban was reviewed with Mrs. Patient. She is aware that her last dose of Rivaroxaban 15mg po bid will be on evening of Day month 2021. Starting the next day, she will switch to 20mg po daily. The total duration will be determined by her hematologist at the follow-up.
   b. Bleeding & Thrombosis: After reviewing the Oral Anticoagulant Monitoring checklist with Mrs. Patient, no issues were identified. She denies symptoms and signs of major and minor bleeding or new clotting. As mentioned, this was her first thromboembolic event, and she reports no prior history of major bleeding.
   c. Adherence: Mrs. Patient reports not missing any doses of her Rivaroxaban or any of her other medications. Her medications are prepared in blister pack at the pharmacy and delivered to her home.
   d. Notable Drug Interactions: Mrs. Patient denies taking any systemic NSAIDs and antiplatelets and is aware to avoid them while she is taking any anticoagulant.
   e. Relevant labs: Her serum creatinine at hospital discharge was 105 (estimated GFR 45).
   f. OAC education: We reviewed key Rivaroxaban education points relevant to her:

i. Indication and rationale for rivaroxaban – Treatment for new pulmonary embolism, she will require OAC for at least 3-6 months,
ii. The recommendation to take rivaroxaban with a meal to improve absorption,
iii. The importance of adherence, and how to handle missed doses,
iv. Side effects, i.e. minor and major bleeding, with examples of signs and symptoms of both, and the need to seek immediate medical attention if any major bleeding occurs,
v. The need to be informed around the peri-operative management of rivaroxaban for any potential planned/upcoming procedures, and keeping all medical appointments,
i. Plan for bloodwork at least every 6 to 12 months for CBC & renal function to monitor the safety of rivaroxaban.

3. Other Medication-Related Issues:
   a. Mrs. Patient's perindopril was initially held due to a rise in serum creatinine at admission, and restarted on discharge. This will require a follow-up lab work to monitor. Her home BP & HR readings have ranged from SBP: 102 – 112, DBP: 66 – 78, HR: 78-89. Her amlodipine was recently discontinued as her BP remains low-normal while on Perindopril 8 mg po daily.
   b. The dose of Janumet was decreased from 1000/50 mg BID to 500/50 mg BID given her most recent HbA1Cs were approx. 5.6 to 6.5%.
   c. Mrs. Patient has been using Lorazepam 1mg po hs for insomnia for some years. She feels that it helps her relax. We discussed the lack of evidence for lorazepam benefit for sleep, and the considerable evidence of harm including confusion, daytime hangover, driving crashes, dependence, etc. I advised her to try some sleep-hygiene techniques and see if she would be agreeable to develop a plan to taper Lorazepam. She has agreed on decreasing the dose to 0.5mg po hs for 2 weeks until the appointment with you for re-assessment.
      i. If she is tolerating the dose decrease, it would be appropriate to decrease the dose further or discontinue.

4. Upcoming Appointments
   a. Family Doctor – Dr. Family Doctor – Date month, 2021
   b. Upcoming Scheduled Procedures: Xxxx: Xxxx, 2021 (Location: Xxxx)
   c. Specialists:
      i. Dr.Specialist, Hematology, – Day month, 2021
   d. Bloodwork – TBD
   e. COACHeD Study: Day month, 2021

5. MAIN RECOMMENDATIONS:
   a. Please book a follow-up assessment in a couple of weeks to review her lorazepam taper, her blood pressure, her resolving PE, and check on her Rivaroxaban therapy.
   b. Suggest further tapering or stopping the lorazepam.

Please contact us at the email or telephone number below if there are any questions or concerns.

Kind regards,

Xxxxxx Pharmacist, RPh; 905-522-1155 x34618; coached@stjoes.ca for
Dr. Anne Holbrook, MD, PharmD, MSc, FRCPC;
Director, Division of Clinical Pharmacology & Toxicology, McMaster University

Cc: Dr. Specialist 1
Dr Specialist 2
